# Compositional analysis of Swertia chirayita medicinal plant using laser-induced breakdown spectroscopy and ICP-MS

**DOI:** 10.1371/journal.pone.0309647

**Published:** 2024-09-20

**Authors:** Habib ur Rehman, Najam ul Hassan, Mohsan Jelani, Kaseb D. Alanazi, Nasar Ahmed, Tariq Saif Ullah, Muhammad Sufyan Javed, Khaled Fahmi Fawy

**Affiliations:** 1 Department of Physics, University of Kotli Azad Jammu and Kashmir, Kotli, Pakistan; 2 Department of Physics, Division of Science & Technology, University of Education, Lahore, Pakistan; 3 Department of Chemistry, Faculty of Science, University of Hail, Hail, Saudi Arabia; 4 Department of Physics, University of Azad Jammu and Kashmir, Muzaffarabad, Pakistan; 5 Department of Botany, University of Kotli Azad Jammu and Kashmir, Kotli, Pakistan; 6 School of Physical Science and Technology, Lanzhou University, Lanzhou, China; 7 Research Center for Advanced Materials Science (RCAMS), King Khalid University, Abha, Saudi Arabia; 8 Department of Chemistry, Faculty of Science, King Khalid University, Abha, Saudi Arabia; NINAR Nishtar Hospital Multan / COMSATS University Islamabad / Kazan Federal University, PAKISTAN

## Abstract

One of the most significant medicinal plants used to treat numerous illnesses is *Swertia chirayita*. The present study demonstrated the compositional analysis of the *Swertia chirayita* (*S*. *chirayita*) plant using an emerging and non-destructive laser-induced breakdown spectroscopy (LIBS) technique. Mg, Ca, K, Fe, Sr, Cr, and Na were verified as necessary elements by the optical emission investigations, while Al, Ti, Si, Ba, Mn, and Li were non-essential. Using the Boltzmann plot technique with stark broadening parameters, plasma temperature and electron number density were calculated in the range of (10,000–12,000) K ±1000 K and (1.5–1.8) × 10^17^ cm^-3^, respectively. Finally, compositional analysis was carried out using calibration-free (CF-LIBS) analysis and results were compared with ICP-MS. It was observed that the concentration of Ca and Fe is higher than other detected elements. All the toxic elements are found to be within the safe limit. So, this medicinal plant can be used to cure a variety of diseases that arise due to the deficiency of these elements.

## Introduction

*Swertia* belongs to the family Gentianaceae which comprises perennial and annual herbs, having approximately 135 species in it. The species of this genus are commonly used in making herbal medicine curing a variety of disorders. *S*. *chirayita*, common name: “Chiretta” is considered a critically endangered species as it grows at higher altitudes between 1200 to 2100m elevation. This plant grows in the sub-temperate zone of the Himalayas of Kashmir and Bhutan [1] at the highest slopes of moist and shaded soil [2]. Due to the continuous usage of this plant in herbal remedies, it is over-exploited from its natural habitat and it is now on the edge of elimination in the wild. *S*. *chirayita* has been used as a traditional Ayurvedic herb by different indigenous inhabitants in multiple ways to gain benefit from it. The plant as a whole is utilized for the treatment of inflammation, hepatitis, and digestion-related issues [3]. Apart from this, it is also used to cure different types of fever, anaemia, malaria, hepatotoxic disorders, bronchial asthma, liver disorders, constipation, gastritis, skin diseases, dyspepsia, epilepsy, worms, scanty urine, ulcers, melancholia, hypertension, and nervous disorders, blood purification, diabetes and secretion of bile [4]. Chirayita provides us with fresh key compounds for the creation of pharmaceuticals that target various pharmacological targets [5]. It is presently in danger of going extinct in the wild as a result of its extensive usage in traditional medicine, which has led to more exploitation than natural habitats [6].

Different analytical techniques were used for the compositional analysis of *Swertia chirayita*. Kshirsagar [7] analyzed *Swertia chirayita* using the HPLC method and indicated phenolic components. The chemical composition of *chirayita* was also studied by Tabasuum et al. [8]. It was discovered that Amarogentin (chirantin), Amarogentin (chirantin), Gentianine, Swerchirin, Swertiamarin, Xanthones, Mangiferin, Lignan, Triterpenoids, and Pentacyclic Triterpenoids are chemical substances present there.

However, the methods employed so far for the analyses are difficult, pricey, and time-consuming. Further research is necessary to develop a quick, straightforward, and reliable method for compositional analysis of the *Swertia chirayita*. The present study describes the compositional analysis of *Swertia chirayita* using laser-induced breakdown spectroscopy (LIBS). Laser-induced breakdown spectroscopy (LIBS) has been recognized as a rapid and insignificantly damaging tool for an entire compositional analysis of various elements with minute or no-sample preparation as compared to usually accessible techniques of spectroscopy [9]. This technique has potential applications in many fields including deep-sea study, industry, explosives investigation, environmental checking, food, agriculture and medicine [10]. Also, it yields emission spectra of the sample which are under observation which have characteristic spectral lines belonging to the several elements which are present in the targeted material including solids, liquids and gases [11]. It is believed that LIBS is well-suited to manage the variety of real-time measurement requirements in the domains of agriculture and plant sciences because of its advanced features [12].

The current work proposes a fundamental and exact approach that can be used to accurately analyze several components (the root, stem, leaves, and flower) of *Swertia chirayita* as opposed to complicated, expensive, and time-consuming lab methods. To understand how procedure parameters impact the samples, it is crucial to know the operating plasma temperature and electron number density of the LIBS. Target material, laser irradiance, wavelength, pulse duration, atmospheric conditions, and geographic location influence the procedure’s properties [13]. Numerous research has demonstrated that the Boltzmann equation may be used to estimate the temperature that regulates the distribution of energy level excitation using an optically thin LIBS [14, 15], while calculations based on the Saha equation, often known as the plasma equation, can be used to estimate the ionization temperature [16, 17]. The main aim of this study is to check essential, major and traces of elements present in plant parts which are used by common people for the treatment of different diseases by using a relatively simple and fast technique. Due to the negligible or minimal sample preparation, LIBS is able to provide relatively cheap and rapid response for the detection of essential, major and trace elements in medicinal plants. The plant’s composition can inform conservation efforts, such as ex-situ conservation (e.g., seed banks) or cultivation programs. Moreover, the plant may have cultural or traditional significance, and its compositional analysis can help preserve this knowledge for future generations. This study also demonstrates how the LIBS technique can be used to analyze and monitor quality in the pharmaceutical and medicinal plant industries.

## Material and methods

### Sample preparation

The roots, stems, leaves and flowers of the *Swertia chirayita* plant were taken from district Bagh, Azad Jammu and Kashmir, Pakistan for the LIBS analysis. The samples were collected, dried in the sun, and then chopped into extremely small pieces with an average diameter and thickness of 20 mm and 2 mm, respectively. Then, 2 g of pieces were compacted into pellets using a hydraulic press at 25,000 psi of pressure.

### Experimental setup

In the literature, the fundamental experimental design has been explored [18, 19]. This high-power Q-switched Nd: YAG laser, manufactured by Brilliant-B Quantel in France, has a pulse duration of 5 ns, a 200 mJ output at 532 nm, and a 400 mJ output at 1064 nm. By adjusting the Q-switch delay, the laser pulse energy was controlled using an energy meter (Nova-Quantal, France). The surface of the pellet, which was positioned on a revolving platform, was focussed by a green laser with a pulse energy of 50–100 mJ through a quartz convex lens with a 10 cm focal length. The laser’s focussed beam was measured to have a diameter of around 0.1±0.01 cm and fluence was approximately 6–12 Jcm^−2^. The air in front of the sample was stopped from breaking down by keeping it closer to the lens than its focal length. An optical fibre (high-OH, core diameter of approximately 600 μm) with a collimating lens (0–45° field of view) positioned normally to the plasma plume was used to collect emitted radiations. A group of four spectrometers (Avantes, Holland) covering the wavelength range of 230–950 nm and each having a 10 μm slit width were used to measure the radiation that was emitted. The emission signals were corrected using the LIBS software by deducting the dark signal from the actual emission signal. The detector gathered the panel-like light, which it later recorded as intensity data. The optical link was equipped with the LIBS detection device to keep track of the plasma emission. The sample material emission spectra were then thoroughly examined using the NIST atomic database [20].

### Results and discussion

Four samples of *Swertia chirayita* (root, stem, flower, and leaves) were analyzed qualitatively and quantitatively by LIBS in the present study. The emission lines of the chemical elements found in the samples are contained in the LIBS spectra. The plant samples contain Mg, Ca, K, Fe, Sr, Cr, Na, Al, Ti, Si, Ba, Mn, and Li.

### Emission studies

**Fig 1:**
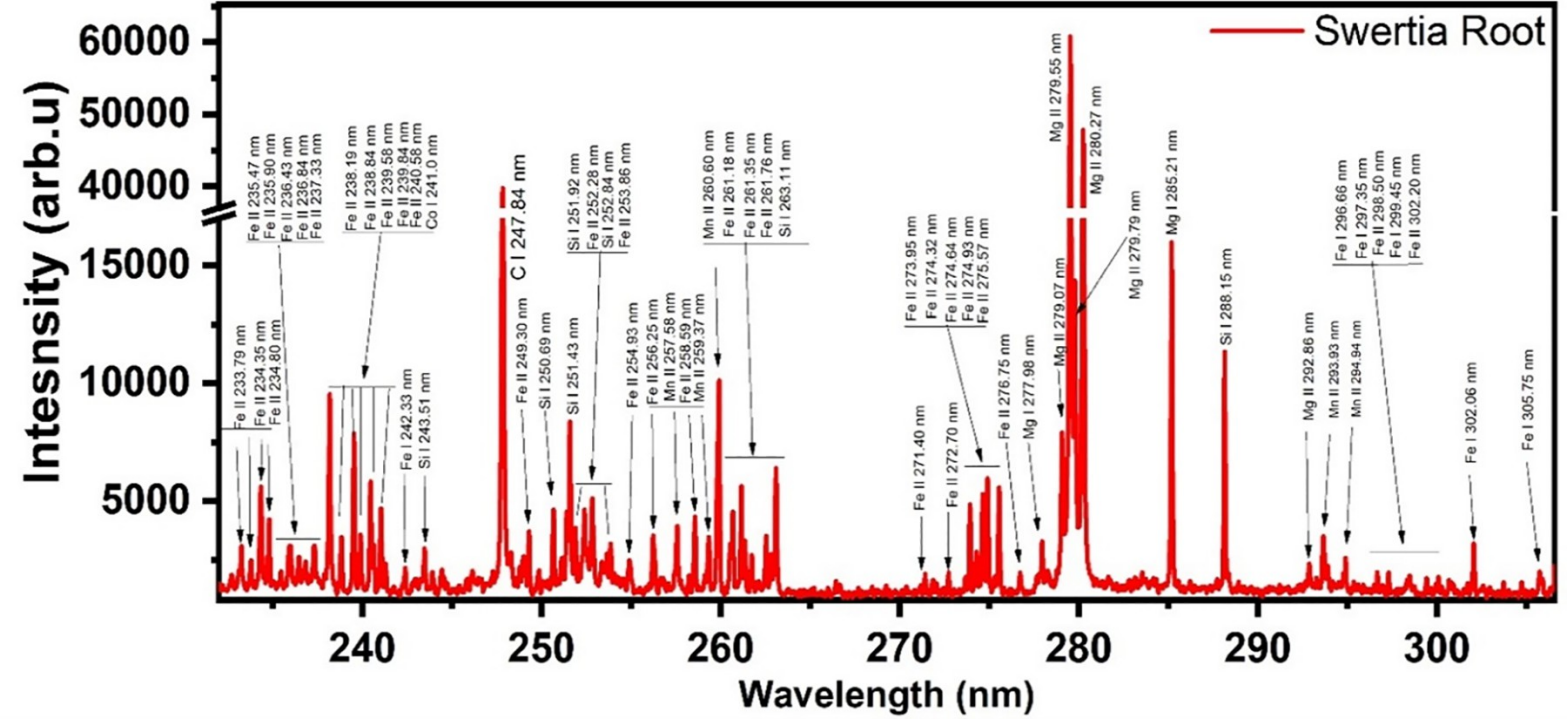
Emission spectrum of roots sample ranging from 230 to 306 nm.

**Fig 2:**
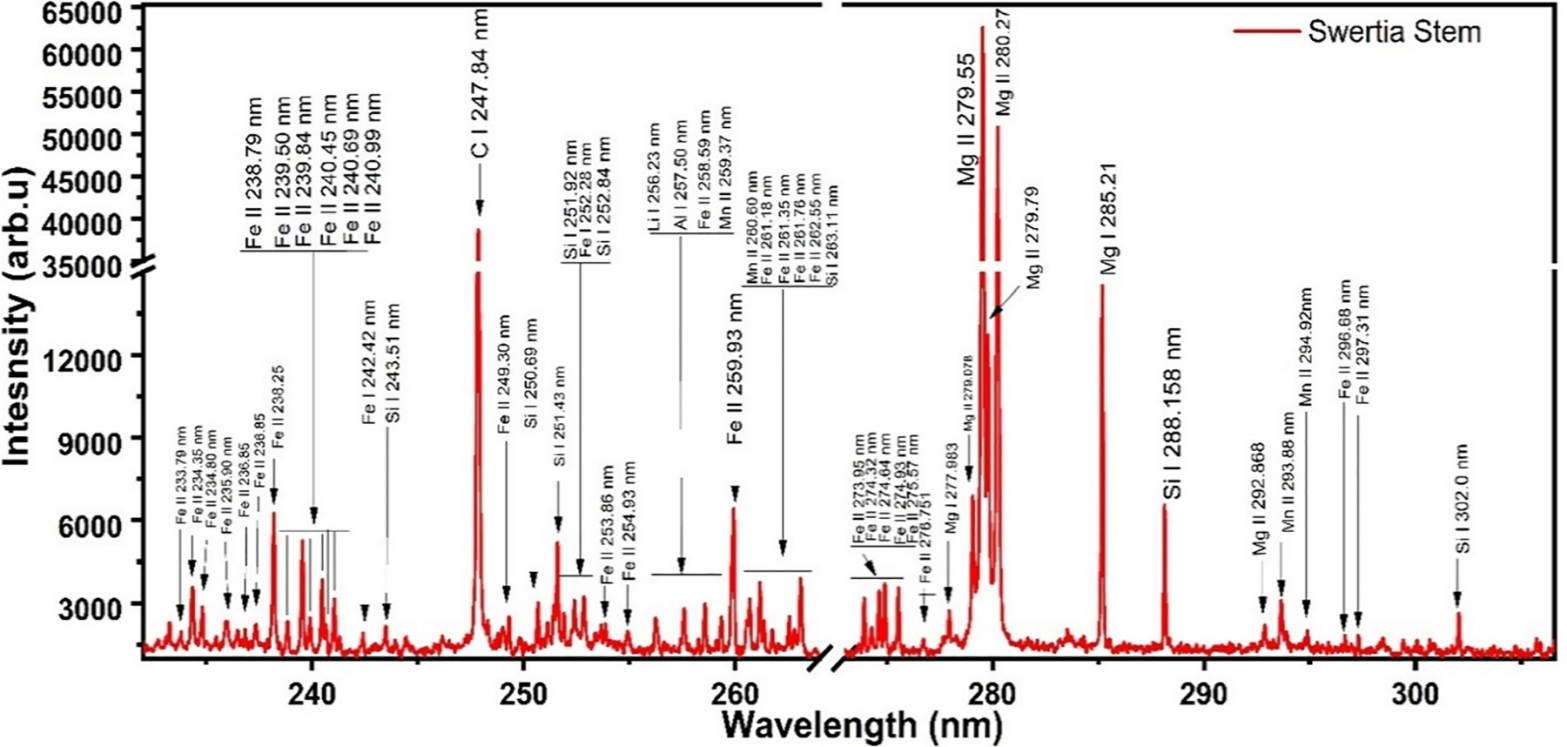
Emission spectrum of stem sample ranging from 230 to 306 nm.

The NIST database’s spectral lines were compared to the emission lines found for each element for the qualitative analysis of all the samples. Recorded emission spectra ranged in wavelength from 240 nm to 880 nm. Four samples show the presence of the emission lines of 13 distinct elements, including Ca, Mg, Fe, Si, Al, Cr, Ti, Ba, K, Li, Sr, Na, and Mn. The emission spectra in the range of 230 to 306 nm are shown in Fig 1. Thirty-one peaks in the spectra were recognized to be for ionized Fe (II). Six lines at 242.33 nm, 296.66 nm, 297.35 nm, 299.45 nm, 302.06 nm, and 305.75 nm are those for neutral Fe (I). Si (I) was found in seven lines at 243.1 nm, 250.59 nm, 251.43 nm, 251.92 nm, 252.84 nm, 263.11 nm, and 288.15 nm. Mg (I) was seen at 277.98 and 285.21 nm, whereas Mg (II) acquired four lines at 279.07, 279.55, 280.27, and 292.86 nm wavelengths. The lines at 257.58, 259.37, 260.60, 293.93, and 294.95 nm were found to be Mn (II). A strong line of C (I) was deducted at 247.84 nm. The second portion of the stem spectrum, with wavelengths ranging from 230 nm to 306 nm, is shown in Fig 2. Numerous lines of Fe (II) that were observed between 230 and 306 nm are present in this spectrum. The spectrum contained the 258.23 nm line of Ti (I). The spectrum revealed five peaks that were recognized as Mg (II), in contrast to neutral Mg (I), which shows two resonance lines at 279.07 nm and 285.21 nm. Seven Si (I) peaks are also seen in the spectrum. Stem sample analysis reveals the presence of calcium, magnesium, aluminium, manganese, iron, titanium, sodium, lithium, potassium, barium, silicon, chromium, and strontium. Magnesium also plays a vital role in the protection of the hepatic cell function as its deficiency has been linked with lipid peroxidation [21].

### Plasma temperature

**Fig 3:**
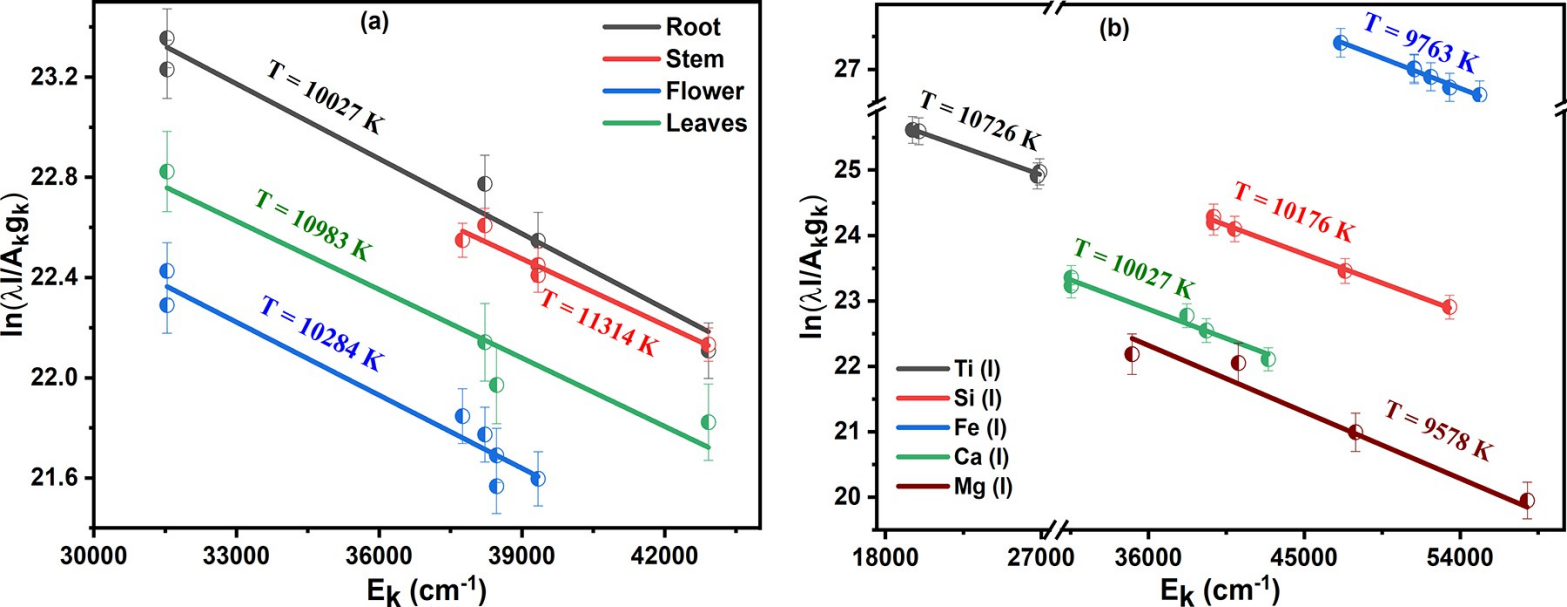
(a) Boltzmann plot for Ca (I) lines of all samples (b) Boltzmann plot for Ti (I), Si (I), Fe (I) Ca (I), and Mg (I) of roots sample.

The plasma may be defined by its temperature (T), which is an important quantity. The plasma temperature was calculated using the Boltzmann plot method [24]. The Boltzmann plot was made using the Ca (I) and Mg (I) lines. Due to experimental irregularities and self-absorption, the data are given with a 10% inaccuracy. The Boltzmann figure was created using the spectroscopic parameters listed in Table 1. Self-absorption is the result of photon absorption in laser-induced plasma. Self-absorption may be considered when the lowest level of transitions is near the ground state. There appears to be a self-reversal drop at the front of the queue when there is a lot of self-absorption. Saturated lines must be avoided while measuring plasma temperature since they cannot provide valuable information. The Boltzmann plot consisting of Ca (I) lines of all samples is shown in Fig 3(A). The average plasma temperature for Ca (I) of all samples is (10652±1000) K. Fig 3(B) shows that the average plasma temperature of Ti (I), Si (I), Fe (I) Ca (I), and Mg (I) of roots sample is to be of (10054±1000) K.

**Table 1:** Spectroscopic parameters of Ca (I), Mg (I) and Si (I) emission lines.

Wavelength λ (nm)		Transition						Transition probability (10^7^s^-1^)		E_k_ (eV)		g_k_	
		Upper Level				Lower Level							
**Ca I**													
610.27		3p^6^4s5s		→		3p^6^4s 4p		0.96		3.91		3	
612.22		3p^6^4s5s		→		3p^6^4s4p		2.87		3.91		3	
560.12		3p^6^3d4p		→		3p^6^3d4s		0.86		4.73		5	
518.88		3p^6^4s5d		→		3p^6^4s4p		4.0		5.32		5	
527.02		3p^6^3d4p		→		3p^6^3d4s		5.0		4.87		5	
**Mg I**													
285.21		3s3p ^3^		→		2p^6^3s^2^		49.1		4.34		3	
383.82		3s3d		→		3s3p		16.1		5.94		7	
277.98		3p^2^		→		3s3p		40.9		7.17		5	
516.73		3s4s		→		3s3p		1.1		17.5		3	
**Si I**													
288.15		3s^2^3p4s		→		3s^2^3p^2^		0.17		5.08		3	
243.51		3s^2^3p3d		→		3s^2^3p^2^		4.43		5.87		5	
251.43		3s^2^3p4s		→		3s^2^3p^2^		7.3		4.52		3	
252.84		3s^2^3p4s		→		3s^2^3p^2^		9.04		4.52		3	
263.11		3s^2^3p3d		→		3s^2^3p^2^		0.1		6.61		3	

### Electron number density

**Fig 4:**
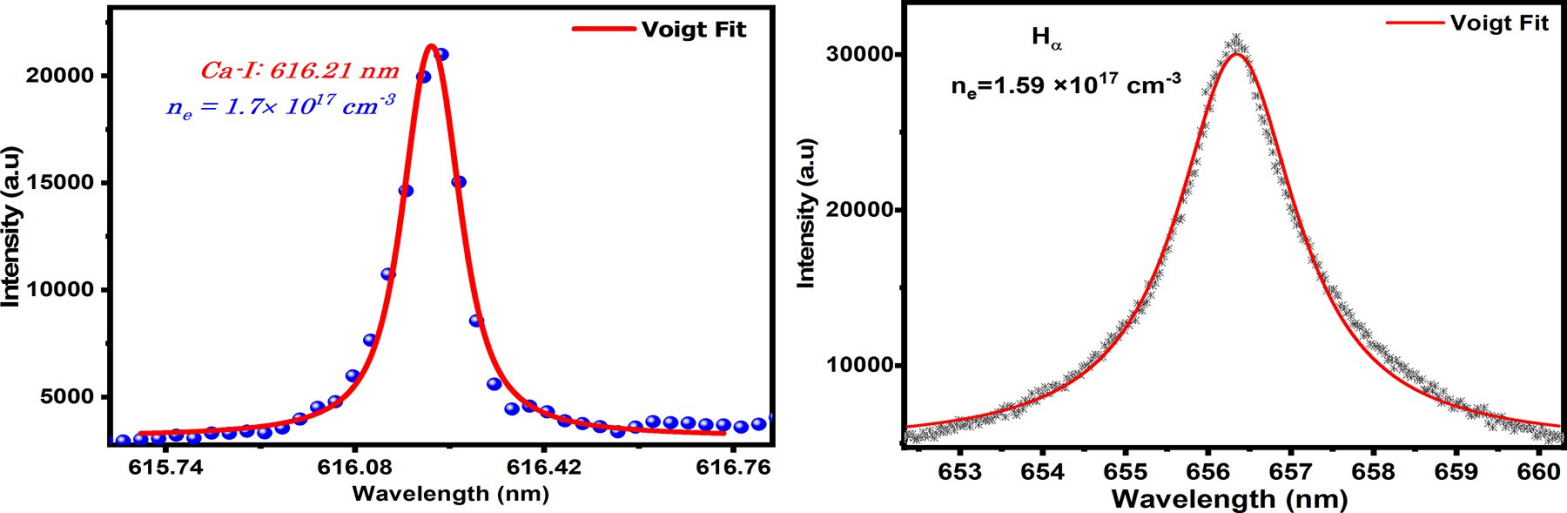
The stark broadened profile of Ca (I) at 616.21 nm and *H*_*α*_ line of roots sample.

The electron number density (*n*_*e*_) was calculated using the full-width half maxima (FWHM) of the well-isolated and sharply widened Ca (I) line at 616.21 nm. The Ca (I) line’s sharp widening profile, with a wavelength of 616.21 nm, is shown in Fig 4. The spectrum was recorded using a Nd: YAG laser that can provide 100 mJ of energy and operates at 532 nm. The experimental data points are represented by the dots in the figure. The value of FWHM is shown by the solid line, which is a Voigt fit. By using Voigt fitting, FWHM for the Ca (I) line at 616.21 nm was calculated to be 0.1097 nm. The following relation was used to estimate the electron number density [22].

$$n_{e}=\frac{{\Delta \lambda }_{FWHM}}{2w_{s}}\times N_{r}$$ (1)

Where Δ*λ*_*FWHM*_ is a stark contribution to the inline profile *w*_*s*_ is the stark broadening parameter. The neutral calcium line’s stark broadening parameter w_s_ has a value of 0.00698 nm at 616.21 nm [23]. *N*_*r*_ is reference electron number density and taken as 10^16^ cm^−3^ for neutral lines [23]. From Eq (1), the estimated electron density is (1.7±0.1)×10^17^ cm^−3^. The likely electron density from *H*_*α*_-line is (1.59±0.1)×10^17^ cm^−3^.

To confirm that the laser-generated plasma is in local thermodynamic equilibrium (LTE), McWhirter [24] proposed criteria for the lower limit of n_e_, given as

ne>1.6×1012×ΔE3×T12 (2)

’ΔE’ indicates the energy differential between the higher and lower energy levels, while ’T’ indicates excitation temperature. The electron density found in Ca (I) emission lines at 616.21 nm was in the range of (1.5–1.7±0.1) × 10^17^ cm^-3^. When utilizing the McWhirter criteria, number densities are calculated that are much lower than when using the Stark broadening parameters in the range (2.2 ± 0.1) × 10^15^ cm^-3^. The electron number density obtained using McWhirter criteria is substantially less than that found by Stark broadening parameters. Therefore, the plasma can be near to LTE.

### Compositional analysis

#### One-line calibration-free LIBS

This method determines the composition of neutral species using the Boltzmann equation. The emission line intensities from the same species are linked by the following equation [25].


$$FC^{z}=l_{k}\frac{U^{z}(T)}{A_{k}g_{k}}e^{\frac{E_{k}}{k_{B}T}}$$ (3)

I_k_ is the line intensity, C_z_ is the number of neutral atoms present, and the "F" factor, which is constant for a given spectral system efficiency, is related to the ablated mass. The factor ’F’ can be computed by correcting the species concentration for normality. This approach makes use of the plasma’s average temperature and electron density. The neutral atom concentration C_z_ is calculated using the equation above using the partition function that is taken from the NIST database at average plasma temperature. The Saha-Boltzmann equation, which links the concentration in a specific element’s two subsequent charge states Z and Z+1, may be used to determine the concentration of the ionized atom C_z+1_ if the ionic lines are absent for certain of the elements.


$$n_{e}\frac{c^{z+1}}{c^{z}}=\frac{{\left(2m_{e}k_{B}T\right)}^{\frac{3}{2}}}{h^{3}}\frac{2U_{z+1}}{U_{z}}\exp\left[\frac{E_{ion}}{k_{B}T}\right]cm^{-3}$$ (4)

Eq (4) provides the ratio of the concentration of two charge states Z and Z+1 of the same elements ($\frac{c^{z+1}}{c^{z}}$) from where by substituting the C^z^ value from Eq (3) for C^z+1^, we can quickly compute its value.

The total concentration of C^a^ and C^b^ is shown as

$$C_{t}^{a}=C_{z}^{a}+C_{z+1}^{a}.C_{t}^{b}=C_{z}^{b}+C_{z+1}^{b}$$ (5)

We utilized the following relationship to determine the percentage compositions:

$$C^{a}\%=\frac{n_{tot}^{a}*(atomic\;weight)}{n_{tot}^{a}*(atomic\;weight)+n_{tot}^{b}*(atomic\;weight)}*100$$ (6)

$$C^{b}\%=\frac{n_{tot}^{b}*(atomic\;weight)}{n_{tot}^{a}*(atomic\;weight)+n_{tot}^{b}*(atomic\;weight)}*100$$ (7)

### Inductively coupled plasma-mass spectrometry (ICP-MS)

Inductively coupled plasma–mass spectrometry (ICP-MS) is used to determine low-elemental concentrations from parts per billion to parts per trillion. The atomic elements are passed through a plasma source, ionised and sorted by their mass-to-charge ratio. This technique has extremely low detection limits, a large linear range, and possibilities to detect the isotope composition of elements [26, 27]. Due to these advantages, the composition of the *Swertia chirayita* was also determined using this technique. The elemental analysis of the *Swertia chirayita* using the CF-LIBS technique was compared with those obtained from the ICP-MS. The results revealed that both methods give similar results which confirmed the reliability of LIBS for determining the elemental composition of *Swertia chirayita*.

Results obtained by CF-LIBS matched with the results obtained using ICP-MS within 5% uncertainty. Table 2 demonstrates the averaged concentration obtained using LIBS and ICP-MS for all the parts of *Swertia chirayita* that were taken from the district Bagh Azad Jammu and Kashmir had two important essential elements, such as Ca and Fe, as well as a moderate amount of one trace element of Si. Almost all of the samples include greater quantities of calcium and iron. Ten other elements like Al, Ba, Sr, Cr, Li, Mg, Mn, K, Na, and Ti are also found.

**Table 2:** Averaged elemental compositions of the several *Swertia chirayita* parts obtained by CF-LIBS and ICP-MS.

Elements	Roots (%)	Stem (%)	Leaf (%)	Flower (%)
Li	0.33	0.52	0.41	0.49
Na	2.25	0.49	0.39	0.83
Mg	5.51	7.37	7.33	6.25
Al	1.57	1.68	1.10	1.58
Si	10.13	8.38	12.60	10.50
K	4.49	2.63	1.21	2.59
Ca	35.51	51.55	28.12	25.91
Sr	2.48	4.34	4.35	5.96
Ba	3.78	2.15	4.75	7.43
Ti	0.59	0.35	0.32	0.49
Mn	0.06	0.07	0.07	0.10
Fe	30.18	15.28	36.18	33.48
Cr	4.85	5.20	3.18	4.42

**Fig 5:**
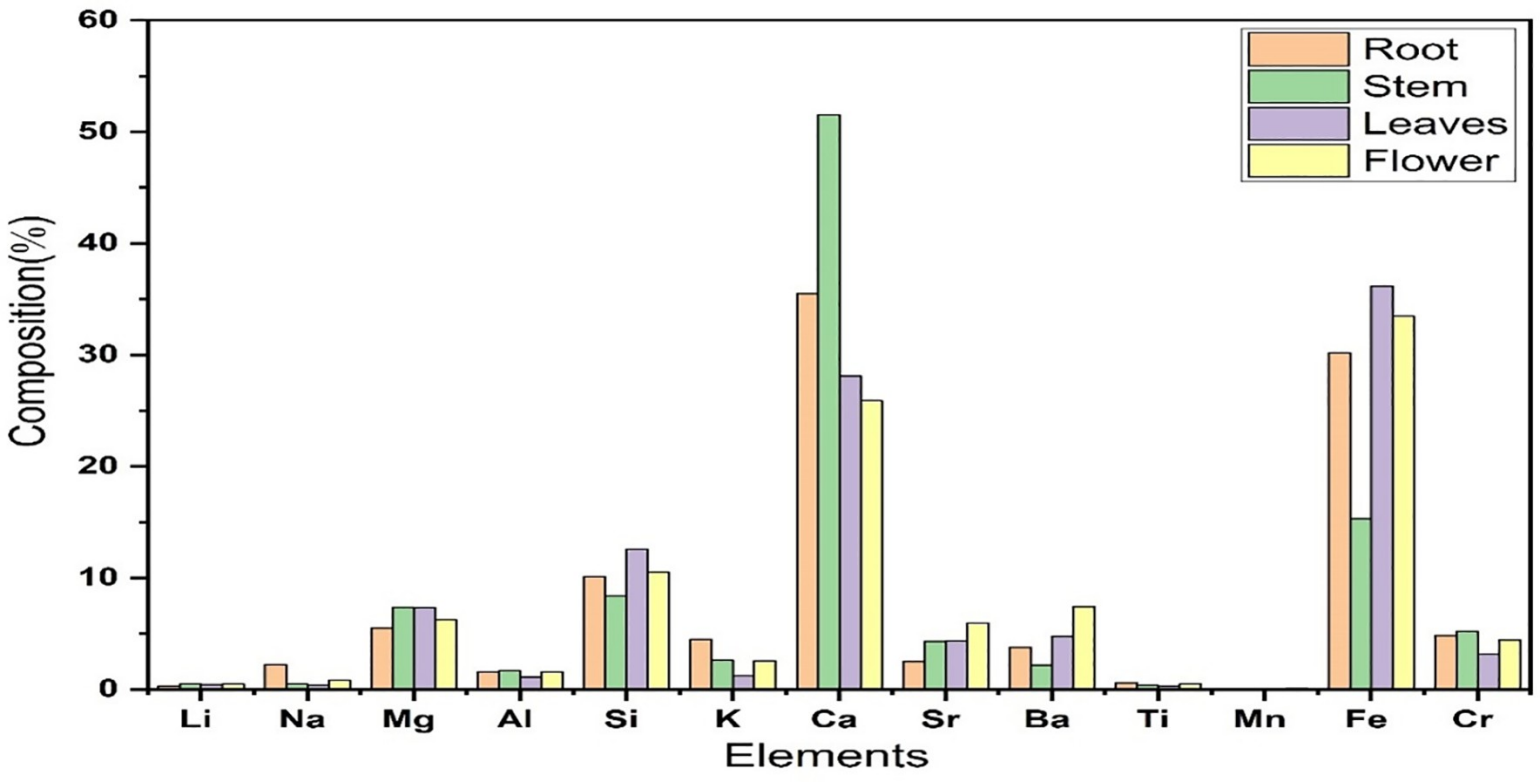
Elemental compositions of various parts of Swertia chirayita.

From Table 2, It is clear that the stem and roots of the plant contain a larger quantity of calcium than the rest of the plant. Additionally, leaves have a somewhat greater calcium content than flowers. According to the findings, calcium predominates in every area of the plant and can be utilized to cure a variety of ailments. It has a substantial impact on how muscles and blood vessels contract and relax [28]. Heart attack, high blood pressure, and osteoporosis brought on by ageing are lessened by it [29]. The concentration of calcium is higher in the root and stem samples than in any other mineral during the investigation. These plant components may thus help treat bone issues as well as the cell communication that regulates muscular contraction and relaxation. According to the findings, leaves and flowers have a higher proportion of iron than roots.While the stem contains a small amount of iron. It may be helpful for different medical issues. It seems that iron, a trace mineral that naturally appears in medicinal plants, is an essential part of human health. Most of the iron in the blood is stored in the bone marrow, muscles, and liver [30]. Iron is a crucial element in red blood cells, which carry oxygen. Without iron, haemoglobin would not be able to bind oxygen, limiting the amount of life-sustaining energy produced [29]. Utilising the plant’s leaves and flowers may improve cellular health and the immune system’s reaction. From Table 2, it can be seen that the stem and leaves contain more concentration of magnesium than other parts of the plant. Numerous illnesses, including migraines, heart disease, stroke, constipation, diabetes, and hypertension, may result from a lack of it [31]. The stem and leaf samples in the current study had greater magnesium levels than the other plant samples, according to the measured magnesium values. So it may be significant for RNA and DNA synthesis and the above diseases. For cells, organs, and tissues to operate properly, potassium, a vital mineral, must be present in the body [32]. It is necessary for the heart, digestion, and contraction of muscles. Additionally, it maintains the heartbeat regularly and improves blood pressure [33]. According to the present study’s observed potassium values, the root samples have greater potassium levels than the other plant samples. The plant’s roots are essential for the contraction of muscles and the beating of the heart. To promote cardiovascular health, recommendations have been made for large reductions in diet sodium intake [34]. Nearly all of the plant samples include a tiny amount of sodium. Therefore, the plant’s samples may play a crucial part in preserving cardiovascular health. After oxygen, silicon is the element that occurs most frequently in nature. Silicon s has been associated with internal nail, hair, and skin structure, general collagen formation, bone mineralization, and bone health, as well as with immune system health, reduced risk for atherosclerosis, and reduced metal deposition in Alzheimer’s disease [35]. The third-most prevalent mineral in our medicinal plant is silicon. Therefore, the plant may play a crucial part in preserving different diseases. It is known that titanium has no biological use in the human body. Since most titanium is not absorbed by the body, there are no health dangers [36]. It was found in plant samples for this investigation in extremely small numbers, and its concentration was below the permitted level. It is not believed that barium is an essential trace mineral. Consuming it in excess can cause high blood pressure, heart damage, digestive problems, muscle paralysis, and even death [37]. In this study, it was detected in tiny amounts in plant samples which is not considered harmful. Aluminum third most rich element in the planet’s crust, is extremely dangerous to all life, including humans [38]. Numerous enzymes, including phosphoxidase, hexokinase, and phosphodiesterase, are inhibited by it [39]. Plant samples have a negligible quantity of it that is within permissible levels. Lithium plays an important role in mood stabilization, antimanic effect, nonspecific reduction of overactivity, anti-aggressive effect, antidepressant effect, neuroleptic augmentations and other related diseases [40]. Its excessive dose consumption may result in unconsciousness, cardiac arrest, nausea, kidney damage, and vision impairment [41]. So it is beneficial for that type of health issue. It is found in the sample in an amount that is far below hazardous thresholds. In the same way, Table 2 demonstrated that in comparison to other measured elements, Sr and Cr concentrations are often low throughout the plant. In conclusion, as represented in Fig 5, a total of thirteen elements in various quantities were found throughout the plant. Ca and Fe are more abundant than any other elements that have been found. It can be seen that the concentration of hazardous elements lies within the safe limit so this plant can be used for various medical purposes.

## Conclusion

The *Swertia chirayita* plant’s composition was examined using non-destructive laser-induced breakdown spectroscopy. The different parts of this plant samples contained samples of the six non-essential elements Al, Ti, Si, Ba, Mn, and Li as well as the seven essential elements Mg, Ca, K, Fe, Sr, Cr, and Na, according to an examination of optical emission spectra. We used the Boltzmann plot method and broadening parameters to get the electron density and plasma temperature. The plasma temperatures of all the plant samples were determined to be in the range of (10,000–12,000) ±1000 K and electron density lies in the range (1.5–1.8) ×10^17^ cm^-3^. The obtained result showed that the measured level of non-essential/toxic components is below the permitted levels. Different parts of plants accumulate numerous elements from the soil so these parts can be used for curing various diseases. It has been observed that the concentration of Ca and Fe is significantly high in all parts of the plant. In conclusion, *Swertia chirayita* could be used as a promising treatment for a variety of illnesses without causing any negative effects on the human body. In addition, this study also shows that the LIBS technique can be effectively utilized for the analysis and quality control of pharmaceutical as well as medicinal plant industries.
